# Clinical Profile and Survival of Oropharyngeal Squamous-Cell Carcinoma in a Region of Northern Spain with a Low HPV-Attributable Fraction: A Retrospective Cohort Study

**DOI:** 10.3390/cancers18213433

**Published:** 2026-10-23

**Authors:** Carlos M. Chiesa-Estomba, Maider Andueza-Guembre, Paulina Droppelman-Álvarez, Laura Basterretxea-Badiola, Leyre González-Vallejo, Pablo Aguirre-Echeverria, Ekhiñe Larruscain-Sarasola, José Ángel González-García, Jon Alexander Sistiaga-Suárez

**Affiliations:** 1 Department of Otorhinolaryngology—Head and Neck Surgery, Hospital Universitario Donostia, Paseo Dr. Begiristain 1, 20014 San Sebastián, Gipuzkoa, Spain; 2 Department of Medical Oncology, Hospital Universitario Donostia, Paseo Dr. Begiristain 1, 20014 San Sebastián, Gipuzkoa, Spain; 3 Department of Radiation Oncology, Hospital Universitario Donostia, Paseo Dr. Begiristain 1, 20014 San Sebastián, Gipuzkoa, Spain; 4 Department of Pathology, Hospital Universitario Donostia, Paseo Dr. Begiristain 1, 20014 San Sebastián, Gipuzkoa, Spain

**Keywords:** oropharyngeal neoplasms, squamous-cell carcinoma of head and neck, human papillomavirus, p16, discordance, survival

## Abstract

**Background/Objectives**: p16 immunohistochemistry is the standard surrogate for HPV causation in oropharyngeal squamous-cell carcinoma (OPSCC), but p16/HPV discordance is more frequent where the HPV-attributable fraction is low. We assessed the p16-positive fraction, p16/HPV-DNA discordance, and survival of OPSCC in northern Spain. **Methods**: Retrospective cohort of 152 consecutive OPSCC (2019–2024). HPV-DNA PCR was performed selectively in p16-positive tumours; selection was assessed with a quantitative bias analysis. Overall survival (OS) was analysed with Kaplan–Meier and Cox regression. **Results**: Fifty-seven tumours (37.5%) were p16-positive; these patients were younger, drank less often, and more often had tonsil/base-of-tongue primaries. HPV-DNA was negative in six of twenty-eight tested p16-positive tumours (discordance 21.4%; 95% CI 10.2–39.5). Testing favoured never-drinkers, and sensitivity analyses for selective testing gave estimates of 16–36%. Two-year OS was 86.3% for p16-positive and 53.9% for p16-negative disease. p16 negativity was associated with death after adjustment for stage, alcohol, and age (HR 3.00; 95% CI 1.44–6.26), but the association was attenuated in curatively treated patients (HR 2.30; 95% CI 0.99–5.33). **Conclusions**: In this region, about one in three OPSCC is p16-positive and one in five tested p16-positive tumours lacks HPV-DNA. Systematic dual p16/HPV testing is advisable before p16 status guides staging or de-escalation.

## Simple Summary

Some cancers of the oropharynx (the back of the throat, including the tonsils and the base of the tongue) are caused by the human papillomavirus (HPV) and have a better outlook than those caused by tobacco and alcohol. Hospitals usually identify HPV-related tumours with a laboratory stain called p16, but this stain can be misleading in regions where HPV-related cancer is less common, such as Spain. We reviewed 152 patients diagnosed in San Sebastián between 2019 and 2024. About one in three tumours was p16-positive, and these patients lived longer. However, when the virus itself was looked for, about one in five p16-positive tumours tested showed no HPV, and because not every tumour was tested, the true figure is uncertain. In regions like ours, p16 should be combined with a direct HPV test before it is used to guide staging or less-intensive treatment.

## 1. Introduction

Head and neck squamous-cell carcinoma remains among the most frequent malignancies worldwide, and oropharyngeal squamous-cell carcinoma (OPSCC) is the subsite whose incidence has risen most in high-income countries over recent decades, a change largely attributed to human papillomavirus (HPV), mainly HPV16 [1,2,3]. The proportion of OPSCC attributable to HPV, however, varies markedly by region: it exceeds 50–70% in North America and northern Europe, whereas southern Europe has consistently shown the lowest attributable fractions [3,4,5]. In Spain, a population-based series from Girona reported 38.5% p16-positive OPSCC in 2016–2018 [6], and a surgical series from Asturias found HPV-DNA/p16 double positivity in only 3–6% of cases diagnosed between 1990 and 2009 [7].

HPV-associated OPSCC is a distinct entity: patients are younger, have less tobacco and alcohol exposure, tumours arise predominantly in the palatine tonsil and base of tongue, and survival is substantially better [8,9]. This led the eighth edition of the AJCC/UICC TNM classification (TNM-8) to introduce a separate staging system for p16-positive OPSCC [10,11], and several trials have explored treatment de-escalation in this population [12].

p16 immunohistochemistry is inexpensive and widely available, and remains the recommended surrogate marker [13,14]. It is, however, an indirect marker, and a proportion of p16-positive tumours contain no transcriptionally active HPV [15]. The HNCIG-EPIC individual patient data analysis of 7654 patients showed that 10.9% of p16-positive OPSCC were HPV-negative, that this proportion was highest in regions with the lowest HPV-attributable fraction, and that discordant patients had a 5-year OS (54.7%) much closer to p16-negative/HPV-negative (40.4%) than to concordant p16-positive/HPV-positive disease (81.1%) [16]. Dual p16/HPV testing improves prognostic discrimination across TNM-8 stages [17,18], and the 2025 update of the College of American Pathologists guideline now addresses when HPV-specific testing should complement p16 [19].

Contemporary data from low-prevalence areas of southern Europe remain scarce, and in routine practice HPV-specific testing is often requested selectively, which may bias estimates of discordance. Throughout this report, “p16-positive fraction” refers to the proportion of tumours with p16 overexpression, whereas “HPV-attributable fraction” refers to the proportion causally driven by HPV; because p16 is a surrogate, the former cannot be equated with the latter, particularly in low-prevalence regions [5,16]. The description of our region as having a low HPV-attributable fraction therefore relies on external data [5,6,7,16], as the present study measured p16 positivity rather than HPV causation. The aims of this study were: (1) to describe the proportion of p16-positive OPSCC and its temporal trend in a tertiary centre in northern Spain; (2) to compare the clinical profile and staging-independent nodal burden of p16-positive and p16-negative tumours; (3) to estimate p16/HPV-DNA discordance while explicitly assessing selection in who was tested; and (4) to evaluate the independent prognostic value of p16 status for overall survival.

## 2. Materials and Methods

### 2.1. Study Design and Population

We conducted a retrospective observational cohort study in a tertiary referral centre. All consecutive patients with a new histologically confirmed squamous-cell carcinoma of the oropharynx (palatine tonsil, base of tongue, soft palate, tonsillar pillar, and lateral/posterior pharyngeal wall) diagnosed between August 2019 and August 2024 with available p16 immunohistochemistry were eligible. p16-negative carcinomas of unknown primary (TX) were excluded from the main analysis because, under TNM-8, they are not classified as oropharyngeal cancer; they were retained in a sensitivity analysis. Non-squamous oropharyngeal tumours (lymphomas, minor salivary gland tumours, and mesenchymal neoplasms) were excluded; p16 immunohistochemistry was available for all OPSCC diagnosed during the study period. The study was approved by the local Research Ethics Committee (code CCH-0227) and conducted in accordance with the Declaration of Helsinki; the requirement for informed consent was waived because of the retrospective design and the use of pseudonymised data. The report follows the STROBE statement [20].

### 2.2. Variables

Data were extracted from clinical records: age, sex, tobacco (current, former, never) and alcohol use (current, former, never), primary subsite, clinical TNM-8 stage, p16 status, HPV-DNA PCR result and genotype, primary treatment (radiotherapy alone, chemoradiotherapy, transoral surgery, open surgery, systemic therapy alone, or palliative/best supportive care), dates of diagnosis, last follow-up and death, recurrence, distant metastasis during follow-up, and second primary tumours. Alcohol use was recorded as reported in the clinical history; drinking status was classified according to the World Health Organization definitions as current (any alcohol consumption in the previous 12 months), former (previous consumption but none in the previous 12 months) or never (lifetime abstainer) [21]. Comorbidity indices (e.g., Charlson or ACE-27) and ECOG performance status were not recorded in the study database. Second primary tumours were defined according to the Warren and Gates criteria (a histologically confirmed malignancy distinct from the index tumour, in which metastasis of the index tumour had been excluded). Patients with pulmonary lesions were referred to the Chest Cancer Clinic for bronchoscopy, endobronchial ultrasound-guided sampling, or biopsy; a pulmonary lesion with squamous-cell histology was usually classified as a metastasis of the index tumour rather than a second primary. The timing of second primaries relative to the index tumour (synchronous vs. metachronous) was not recorded in the database. Survival time was measured from diagnosis to death or last recorded contact. The latest recorded contact was in July 2026, but follow-up was not updated to a common cut-off date for all patients; vital status was ascertained from each patient’s electronic medical record. Missing data were not imputed. Because TNM-8 uses different N definitions for p16-positive and p16-negative OPSCC, nodal burden was additionally compared using descriptors common to both systems: any clinical nodal involvement (cN+), bilateral or contralateral nodal disease (p16-positive cN2; p16-negative cN2c), and any node > 6 cm (p16-positive cN3; p16-negative cN3a). Clinical extranodal extension (cN3b) exists only in the p16-negative classification and is reported descriptively. Clinical extranodal extension was coded on physical examination in the clinic, when the nodal mass was immobile or fixed to the deep planes; radiological or pathological extranodal extension alone was not coded as cN3b.

### 2.3. p16 Immunohistochemistry and HPV Testing

p16 immunohistochemistry was performed with the CINtec p16 Histology monoclonal antibody (E6H4; Roche - Basel, Switzerland) and considered positive when strong, diffuse nuclear, and cytoplasmic staining was present in >70% of tumour cells, according to CAP criteria [13]. HPV-DNA was detected by real-time PCR on DNA extracted from formalin-fixed, paraffin-embedded tumour tissue obtained by biopsy in the clinic, from the surgical specimen or by core-needle (Tru-Cut) biopsy of a cervical lymph-node metastasis; tumour-rich areas were selected on a haematoxylin–eosin section and macrodissected before DNA extraction. Three commercial real-time PCR assays were used during the study period: the cobas HPV Test (Roche Diagnostics - Basel, Switzerland), which reports HPV16 and HPV18 individually and 12 other high-risk types (31, 33, 35, 39, 45, 51, 52, 56, 58, 59, 66, and 68) as a pooled result; the Anyplex II HPV28 Detection assay (Seegene—Seoul, South Korea), which uses dual-priming oligonucleotide (DPO) and TOCE technologies to detect and differentiate twenty-eight genotypes (nineteen high-risk and nine low-risk, including HPV6, HPV35, and HPV54) in a single reaction; and the BD Onclarity HPV assay (Becton Dickinson—Franklin Lakes, NJ, USA), which reports HPV16, 18, 31, 45, 51, and 52 individually and the remaining high-risk types in three groups (33/58, 56/59/66 and 35/39/68); the assay used changed over the study period, and the number of tumours tested with each assay could not be retrieved. These assays were developed and validated for cervical specimens and were not locally validated on oropharyngeal formalin-fixed, paraffin-embedded (FFPE) tissue; their performance on FFPE head and neck carcinomas has been reported with high concordance against reference methods [22,23]. Because HPV16 is reported individually by all three assays, a genotype should be available for every HPV16-positive tumour; however, the genotype was recorded in the study database for only 12 of the 22 HPV-DNA-positive tumours, and for the remaining 10 the genotype result was not available in the institutional records. All three assays include an internal control of human cellular DNA (β-globin in the cobas and Anyplex assays). This control confirms the presence of amplifiable human DNA but not of adequate tumour DNA: a sample rich in lymphocytes or stroma, such as a nodal core, can pass the control and still yield a false-negative HPV result. p16 staining was scored in routine practice by two pathologists with head and neck expertise before any HPV-DNA result was available, because HPV testing was requested after the p16 result. Equivocal cases (strong staining in 50–70% of cells, or weak/patchy staining) were reviewed jointly by both pathologists to reach a consensus. Slides were not centrally re-reviewed for this study, and inter-observer agreement was not formally assessed. HPV testing was not systematic; it was requested at the discretion of the treating team, reflecting the progressive local implementation of the technique. Tumours were classified as concordant (p16+/HPV-DNA+), discordant (p16+/HPV-DNA−), p16-positive not tested, and p16-negative.

### 2.4. Outcomes and Statistical Analysis

The primary outcome was overall survival (OS), defined as time from diagnosis to death from any cause, censoring alive patients at last contact. Because follow-up was not updated to a common date and 47 of the 54 deaths were identified in the electronic record after the last clinical contact, two sensitivity analyses with administrative censoring were performed: (i) all patients were censored at 31 October 2024, the period in which most surviving patients had their last recorded contact, and deaths after that date were not counted; and (ii) surviving patients were censored at 31 July 2026, the date by which vital status had been verified for all patients by review of each patient’s electronic medical record. Median follow-up was estimated with the reverse Kaplan–Meier method.

Continuous variables are reported as mean ± standard deviation (range) and compared with Student’s *t* test after checking normality (Shapiro–Wilk); categorical variables are reported as *n* (%) and compared with Fisher’s exact test (2 × 2 tables) or the χ^2^ test, using a Monte Carlo exact permutation test when >20% of expected counts were <5. Proportions are given with Wilson 95% confidence intervals (CI). Recurrence, distant metastasis and second primary tumours are reported as crude proportions without hypothesis tests, because follow-up and competing mortality differed between groups and event dates were not available for a competing-risks analysis. The temporal trend in the p16-positive proportion was assessed by logistic regression on calendar year. To assess selection in HPV testing, tested and untested p16-positive patients were compared. To bound discordance among all p16-positive tumours, a quantitative bias analysis was performed. First, deterministic bounds were obtained by assuming that all untested tumours were concordant (lower bound) or discordant (upper bound). Second, assuming that HPV testing was missing at random conditional on current smoking—the only characteristic associated with both selection for testing and discordance for which stratum-specific discordance could be estimated—discordance observed among tested patients within each smoking stratum (current vs. former/never) was applied to untested patients of the same stratum; this standardisation is equivalent to inverse-probability-of-testing weighting with smoking as the weighting variable. Uncertainty was propagated by Monte Carlo simulation (200,000 draws from Jeffreys beta posteriors of stratum-specific discordance), and the 2.5th–97.5th percentiles are reported as a 95% simulation interval. Third, inverse-probability-of-testing weights were derived from a logistic model including current smoking and never-drinking, the characteristics most strongly associated with testing, with a 95% CI from 4000 nonparametric bootstrap resamples. Because HPV testing was introduced progressively, this model was repeated adding calendar year of diagnosis. Fourth, a scenario analysis varied the discordance assumed among untested tumours from 10.9% (the pooled HNCIG-EPIC estimate [16]) to 50%.

OS was estimated using the Kaplan–Meier method with log–log 95% CIs, and compared with the log-rank test; curves show censoring marks and numbers at risk. Cox proportional hazards regression was used for univariable and multivariable analyses. The multivariable model was specified a priori with p16 status, T category (T3–T4 vs. T0–T2), N category (N2–N3 vs. N0–N1), alcohol exposure, and age (per 10 years), limiting the number of covariates to keep approximately ≥10 events per parameter. Because age is a strong determinant of non-cancer mortality and differed between groups, the model was repeated without age, and in a sensitivity analysis restricted to M0 patients treated with curative intent. With 35 deaths, the curative-intent models had 5.8–7.0 events per parameter, below the ≥10 criterion, and were considered exploratory. Unspecified subsites were grouped with other subsites in the dichotomised subsite variable and, in a sensitivity analysis, treated as missing. Because TNM-8 T and N categories are defined differently for p16-positive and p16-negative OPSCC, the model was also repeated replacing system-specific N2–N3 with the staging-independent nodal descriptors defined above (any cN+ and bilateral/contralateral nodes; nodes > 6 cm could not be included because no deaths occurred among the five patients with this feature, and clinical extranodal extension is not recorded in the p16-positive classification). Within the curative-intent cohort, the model was further adjusted for, and in a separate analysis stratified by, primary treatment modality (surgery-based vs. non-surgical). The proportional-hazards assumption was checked with Schoenfeld residuals; model discrimination is reported with Harrell’s C-index. A two-sided *p* < 0.05 was considered significant. Given the small number of discordant cases, combined p16/HPV-DNA groups are described only with counts and proportions of deaths (Wilson 95% CI); no survival curves or hypothesis tests were performed for these groups. Analyses were performed in Python 3.13 (pandas 3.0, NumPy 2.5, SciPy 1.18, Matplotlib 3.11). Kaplan–Meier and reverse Kaplan–Meier estimation, the log-rank test, Cox regression (Newton–Raphson, Efron method for ties), the Grambsch–Therneau test of scaled Schoenfeld residuals, Harrell’s C-index, and logistic regression were implemented in custom scripts, whose output was cross-checked against published reference results of the R survival package (version 3.8-6) for a standard dataset (the Freireich leukaemia trial), reproducing exactly the Efron and Breslow coefficients, the standard error, the log-rank statistic, and the Kaplan–Meier estimates. The scripts are available from the corresponding author on reasonable request.

## 3. Results

### 3.1. Cohort and p16-Positive Fraction

The database contained 164 unique patients; 12 p16-negative carcinomas of unknown primary were excluded, leaving 152 patients for analysis. Apart from these cases, no patient with OPSCC was excluded. During the study period, 190 oropharyngeal tumours were diagnosed; twenty-six non-squamous tumours (seventeen lymphomas, six minor salivary gland tumours, and three mesenchymal neoplasms) were excluded before the final database was built (Figure 1). Mean age was 66.3 ± 8.7 years (range 42–87) and 99 (65.1%) were men. Fifty-seven tumours (37.5%; 95% CI 30.2–45.4) were p16-positive. The p16-positive proportion showed no evidence of change over the study period (10/22 [45.5%] in 2019–2020, 10/30 [33.3%] in 2021, 11/25 [44.0%] in 2022, 14/36 [38.9%] in 2023, and 12/39 [30.8%] in 2024; OR per year 0.91, 95% CI 0.72–1.14; *p* = 0.40). The single 2019 case was pooled with 2020.

### 3.2. Clinical Profile According to p16 Status

Patient and tumour characteristics are shown in Table 1. p16-positive patients were younger (63.6 ± 8.4 vs. 67.9 ± 8.4 years; *p* = 0.003), with no difference in sex distribution. Never-drinkers were more frequent among p16-positive patients (63.2% vs. 33.7%; never vs. ever, *p* < 0.001; *p* = 0.001 for the three-category comparison in Table 1), while the overall distribution of tobacco exposure did not differ significantly (three categories, *p* = 0.060); current smoking was less frequent among p16-positive patients (42.1% vs. 61.1%; *p* = 0.029), whereas the difference in never-smokers was not significant (28.1% vs. 15.8%; *p* = 0.095); notably, 71.9% of p16-positive patients were current or former smokers. p16-positive tumours arose more often in the tonsil or base of tongue (87.7% vs. 61.1%; *p* < 0.001; 94.3% vs. 64.4% when the nine unspecified subsites were treated as missing) and never in the soft palate (0% vs. 18.9%).

p16-negative tumours were more frequently T3–T4 (60.0% vs. 35.1%; *p* = 0.004) (Table 2). Using staging-independent descriptors, clinical nodal involvement was more frequent in p16-positive disease (89.5% vs. 70.2%; *p* = 0.008), as was bilateral or contralateral nodal disease (33.3% vs. 12.6%; *p* = 0.003), while nodes > 6 cm were uncommon in both groups (7.0% vs. 1.1%; *p* = 0.066). Clinical extranodal extension (cN3b) was recorded in 24 p16-negative patients (25.3%). Treatment differed between groups (*p* = 0.003): transoral surgery was used more often in p16-positive patients (40.4% vs. 21.1%), followed by adjuvant radiotherapy or chemoradiotherapy in 15/23 (65.2%) and 11/20 (55.0%) patients, respectively, and palliative care alone only in p16-negative patients (13.7%).

### 3.3. p16/HPV-DNA Concordance and Selection in Testing

HPV-DNA PCR was performed in 28 of 57 p16-positive tumours (49.1%): twenty-two were positive and six negative, a discordance rate of 21.4% (95% CI 10.2–39.5) (Table 3). Genotype was recorded for 12 of the 22 HPV-DNA-positive tumours (see Section 2): HPV16 in ten (one co-infected with HPV6), HPV35 in one and HPV54 in one. Because HPV54 is a low-risk type, the tumour-harbouring-only HPV54 may not be HPV-driven; if it is reclassified as HPV-negative, discordance becomes 7/28 (25.0%; 95% CI 12.7–43.4). Three p16-negative tumours were also tested, during the implementation of HPV testing in the pathology department, and were HPV-DNA-negative. Of the four p16-positive T0 tumours, one was tested and was HPV-DNA-positive (HPV35); the other three were not tested.

Tested and untested p16-positive patients differed: tested patients were more often never-drinkers (82.1% vs. 44.8%; *p* = 0.006) and, non-significantly, more often never-smokers (39.3% vs. 17.2%; *p* = 0.082), younger (61.8 vs. 65.4 years; *p* = 0.107), and less often T3–T4 (25.0% vs. 44.8%; *p* = 0.167); all three M1 p16-positive patients were untested (Table 3). The proportion of p16-positive tumours tested increased over the study period (5/10 in 2019–2020, 1/10 in 2021, 6/11 in 2022, 8/14 in 2023, and 8/12 in 2024; OR per year 1.40, 95% CI 0.95–2.07). Four of the six discordant patients were current smokers, compared with three of twenty-two (13.6%) concordant patients, three discordant tumours arose in the palatine tonsil, one in the base of tongue, one in the pharyngeal wall and one in an unspecified subsite. Table 4 details each discordant tumour and the HPV54-only tumour.

Current smokers accounted for seven of twenty-eight tested (25.0%) and seventeen of twenty-nine untested (58.6%) p16-positive patients (*p* = 0.016). Discordance was 57.1% (4/7; 95% CI 25.0–84.2) among tested current smokers and 9.5% (2/21; 95% CI 2.7–28.9) among tested former/never smokers. Deterministic bounds for discordance among all 57 p16-positive tumours ranged from 10.5% (6/57; all untested tumours concordant) to 61.4% (35/57; all untested tumours discordant). Standardised to the smoking distribution of the whole p16-positive group, estimated discordance was 37.4% among untested tumours and 29.6% (95% simulation interval 14.9–45.5) among all p16-positive tumours. In the scenario analysis, overall discordance was 16.1% if untested tumours were discordant at the HNCIG-EPIC rate (10.9%), 21.4% if at the rate observed among tested patients, 25.8% at 30%, and 36.0% at 50%. Discordance was 17.4% (4/23) among tested never-drinkers and 40.0% (2/5) among tested ever-drinkers; standardised to drinking status, estimated overall discordance was 25.7%. With inverse-probability-of-testing weights based on smoking and drinking (odds ratios for being tested 0.35 for current smokers and 4.11 for never-drinkers), estimated discordance was 31.0% (bootstrap 95% CI 6.8–59.4). Adding calendar year of diagnosis to the model gave 28.9% (bootstrap 95% CI 6.8–57.1). These analyses use the primary classification; reclassifying the HPV54-only tumour (from a current smoker and drinker) as HPV-negative increased the smoking-standardised estimate to 35.6%. Taken together, the sensitivity analyses gave estimates of 16–36%; because they rest on small strata, the observed 21.4% (95% CI 10.2–39.5) is regarded as the primary estimate and these values as a sensitivity range.

### 3.4. Outcomes and Survival

Recurrence was recorded in 8.8% of p16-positive and 27.4% of p16-negative patients, distant metastasis during follow-up in 1.8% and 7.4%, and second primary tumours in 15.8% and 37.9%, respectively (Table 1). The curative-intent cohort comprised 126 M0 patients (53 p16-positive and 73 p16-negative; of the 54 p16-positive patients who received potentially curative treatment, one had M1 disease at diagnosis and was excluded) (Figure 1); recurrence occurred in three of fifty-three (5.7%) p16-positive and twenty-one of seventy-three (28.8%) p16-negative patients. Second primary tumours (n = 45) involved the lung in fourteen patients, the head and neck or upper aerodigestive tract in ninetten, the oesophagus in two, and other sites in fourteen (liver four, prostate three, and stomach, kidney, cervix/ureter, bladder, skin, breast, and colon in one each); some patients had more than one, and the site was not recorded in one. These crude proportions do not account for differences in follow-up or competing mortality and should be interpreted cautiously.

With a median follow-up of 28.1 months (reverse Kaplan–Meier; range 0–53.6), 54 patients died (10 p16-positive, 44 p16-negative). OS was higher in p16-positive patients (log-rank *p* < 0.001; Figure 2; Table 5): 1-, 2- and 3-year OS were 91.9% (95% CI 79.7–96.9), 86.3% (71.6–93.7) and 72.2% (53.4–84.4) for p16-positive versus 76.0% (65.5–83.7), 53.9% (41.2–65.1) and 36.3% (23.5–49.2) for p16-negative disease. Median OS was not reached in p16-positive and was 24.9 months in p16-negative patients.

In an exploratory descriptive analysis of combined p16/HPV-DNA status, one of twenty-two concordant p16+/HPV-DNA+ patients died (4.5%; 95% CI 0.8–21.8), compared with two of six discordant p16+/HPV-DNA− patients (33.3%; 95% CI 9.7–70.0) and seven of twenty-nine untested p16-positive patients (24.1%; 95% CI 12.2–42.1); median follow-up (reverse Kaplan–Meier) was 12.9, 28.5 and 36.0 months, respectively. These are crude proportions that do not account for differences in follow-up. Given the very small number of discordant patients and events, the groups were not compared formally and no survival curves are presented; the widely overlapping confidence intervals show that no conclusion about the prognosis of discordant tumours can be drawn from our data alone, and these figures should be regarded as hypothesis-generating only.

In univariable Cox analysis, p16 negativity (HR 3.56; 95% CI 1.78–7.12), older age, T3–T4, and M1 at diagnosis were associated with worse OS; subsite outside the tonsil/base of tongue was of borderline significance (HR 1.74; 95% CI 1.00–3.01) and not significant when unspecified subsites were treated as missing (HR 1.66; 95% CI 0.90–3.06) (Table 6). In the multivariable model, p16 negativity remained independently associated with death (HR 3.00; 95% CI 1.44–6.26; *p* = 0.003), together with T3–T4 (HR 1.96; 95% CI 1.09–3.52; *p* = 0.025); N2–N3, alcohol exposure, and age (HR 1.34 per 10 years; *p* = 0.078) were not significant (C-index 0.70). Excluding age from the model did not materially change the estimate for p16 (HR 3.20; 95% CI 1.54–6.64), whereas restricting the analysis to 125 M0 patients treated with curative intent attenuated it (HR 2.30; 95% CI 0.99–5.33; *p* = 0.052; 35 deaths, 7.0 events per parameter); one p16-negative patient of the one-hundred and twenty-six in this cohort lacked N category. No violation of proportional hazards was detected for the covariates in these models. Including the 12 p16-negative unknown-primary cases gave consistent results (HR 3.78; 95% CI 1.90–7.50). When system-specific N2–N3 was replaced by the staging-independent nodal descriptors, the HR for p16 negativity was 2.97 (95% CI 1.42–6.18; *p* = 0.004), and 3.09 (1.49–6.42) when T4 of either system was used instead of T3–T4. In the curative-intent cohort, further adjustment for treatment modality gave an HR of 2.23 (0.95–5.24; *p* = 0.065), and stratification by treatment modality 2.09 (0.90–4.86; *p* = 0.089). Modelling alcohol in three categories gave HRs of 0.87 (0.47–1.61) for current and 1.04 (0.42–2.57) for former versus never drinkers, without change in the p16 estimate (HR 2.97). The global Schoenfeld test was not significant (*p* = 0.86). With administrative censoring at 31 October 2024 (42 deaths), the adjusted HR for p16 negativity was 4.45 (95% CI 1.78–11.15); censoring surviving patients at 31 July 2026 (54 deaths; median follow-up 39.9 months) gave an HR of 2.51 (95% CI 1.21–5.22; *p* = 0.013), with 2-year OS of 89.5% and 63.3% for p16-positive and p16-negative disease, respectively.

## 4. Discussion

In this contemporary cohort from a region previously reported to have a low HPV-attributable fraction [5,6,16], 37.5% of OPSCC were p16-positive, with no evidence of change over the study period. Among p16-positive tumours tested for HPV-DNA, about one in five was HPV-DNA-negative. p16-positive patients showed the expected clinical profile and markedly better survival, and p16 negativity was associated with a roughly threefold higher hazard of death after adjustment for stage, alcohol, and age.

Our p16-positive fraction is almost identical to the 38.5% reported by the population-based Girona registry for 2016–2018 [6], and far below the proportions seen in North America and northern Europe [3,4]. It is, however, considerably higher than the HPV-driven fraction reported in Asturias two decades ago [7], although that series required HPV-DNA positivity and included only surgically treated patients, so the figures are not directly comparable. The absence of a trend over five years should be interpreted cautiously given the short period and the annual sample size. Importantly, because p16 overestimates HPV causation where the attributable fraction is low [5,16], the true HPV-driven fraction in our area is probably lower than 37.5%.

The observed discordance of 21.4% is numerically higher than the 10.9% pooled estimate of HNCIG-EPIC, although our 95% CI includes that value; it is consistent with its finding that discordance is highest in regions with low HPV-attributable fractions, including Spain [16]. In its only southern European cohort (Barcelona), 28 of 95 p16-positive tumours (29.5%) were HPV-negative [16]. Our estimate, though, must be regarded as partial and potentially biased rather than a population value. HPV testing was selective, and tested patients were more often never-drinkers and never-smokers—the classic HPV phenotype. If testing was preferentially requested in “typical” cases, discordance among untested p16-positive patients, who more often drank and smoked, may be higher still; the wide confidence interval (10.2–39.5%) reflects this uncertainty. Our bias analysis quantifies this concern. Assuming that testing was missing at random within smoking strata, discordance among all p16-positive tumours was estimated at 29.6%, and even the most favourable scenario—untested tumours discordant at the HNCIG-EPIC rate—yielded 16%. Weighting for smoking and drinking gave a similar estimate (31.0%), as did adding calendar year of diagnosis (28.9%). These estimates rely on few variables and small strata, and the deterministic bounds (10.5–61.4%) show that the data alone cannot exclude considerably higher values. With respect to selection alone, they suggest that the observed 21.4% is more likely an under- than an overestimate; conversely, false-negative PCR results would inflate discordance, whereas counting the HPV54-only tumour as concordant would deflate it (25.0% if reclassified), so the overall direction of bias remains uncertain. For these reasons, we regard the observed 21.4% (95% CI 10.2–39.5) as our primary estimate and the bias-adjusted values (16–36%) as a sensitivity range rather than a corrected figure. Retrospective HPV testing of the archived blocks of the 29 untested p16-positive tumours, ideally with an RNA-based assay, would remove the selection problem; this was not feasible within the present study because of current workforce constraints in the molecular pathology laboratory, and remains a recommendation for future work; testing the 17 untested current smokers would most directly address the central uncertainty. Consistent with published data [16], discordant tumours in our series were more frequent in current smokers (four of six vs. three of twenty-two concordant tumours).

p16 overexpression without HPV-DNA may result from HPV-independent mechanisms such as alterations in the RB/CDKN2A pathway, or from false-negative molecular testing due to low tumour content or DNA degradation in archival material [15,24]. Because discordance is defined by two imperfect tests, part of it may reflect measurement rather than biology: p16 interpretation around the 70% threshold can vary between observers [13], and DNA PCR on archival tissue may fail with low tumour cellularity. In our study, p16 was scored in routine practice without central re-review or formal assessment of inter-observer agreement, so we cannot exclude that some of the six discordant cases reflect assay or interpretation variability; blinded re-review and confirmation with an RNA-based assay (E6/E7 mRNA or high-risk HPV RNA in situ hybridisation) would be required to classify them definitively. Table 4 provides the available case-level details; in the five node-positive discordant tumours, testing was performed on ultrasound-guided biopsies of the cervical adenopathy and in the remaining two on biopsies of the primary tumour obtained in the clinic under local anaesthesia, whereas tumour cellularity, assay, and p16 staining pattern could not be retrieved for individual cases, and because the internal control of these assays confirms amplifiable human DNA but not adequate tumour DNA, false-negative results in lymphoid- or stroma-rich samples cannot be excluded. Whatever the mechanism, discordant tumours in large series behave clinically closer to HPV-negative disease: 5-year OS of 54.7% versus 81.1% for concordant cases in HNCIG-EPIC [16]. Our exploratory data (two deaths among six discordant patients versus one among 22 concordant patients) are directionally consistent with this but are too few to support any prognostic statement. Taken together with the external evidence, they reinforce the recommendation that dual p16/HPV testing be performed at least in p16-positive OPSCC in low-prevalence regions [16,17,18,19], particularly in smokers and non-tonsillar/base-of-tongue subsites, and in all cases before considering de-escalation [12,18]. This recommendation has a further implication. p16-negative/HPV-positive tumours are also recognised and, in HNCIG-EPIC, had a 5-year OS of 53.2%, intermediate between concordant p16+/HPV+ and p16−/HPV− disease [16]. A strategy that restricts HPV testing to p16-positive tumours, as in our practice, cannot detect this group; a truly systematic dual-testing approach should therefore also include p16-negative OPSCC, at least those with a suggestive phenotype (never or light smokers, tonsil/base-of-tongue primaries or cystic nodal metastases).

The clinical profile of our p16-positive patients—younger age, less alcohol use, tonsil/base-of-tongue predominance and smaller primaries with more extensive nodal disease—mirrors that described internationally [8,25,26]. The lack of a significant difference in tobacco exposure, with more than 70% of p16-positive patients being current or former smokers, is noteworthy and in line with southern European series [6]. This high smoking prevalence probably contributes both to discordance and to the risk of second primary tumours, which were frequent in our cohort (37.9% of p16-negative patients), and which are a leading cause of death in survivors of head and neck cancer [27]. The rate of second primaries in p16-negative patients is nevertheless high for a median follow-up of 28 months. Because pulmonary lesions with squamous histology were usually classified as metastases, misclassification of lung metastases as second primaries is less likely, although it cannot be excluded; the absence of systematic data on the timing of second primaries also prevents distinguishing synchronous from metachronous tumours.

We deliberately avoided comparing TNM N categories between groups, because p16-positive and p16-negative OPSCC are staged with different N definitions and the same nodal burden may receive different categories [10,11]. For the same reason, the system-specific N covariate in our pre-specified Cox model does not represent identical nodal burden in both groups; replacing it with staging-independent descriptors did not materially change the estimate for p16 (HR 2.97). Using descriptors shared by both systems, p16-positive tumours more often had nodal disease and more often bilateral/contralateral nodes, a pattern consistent with the cystic, multilevel nodal presentation typical of HPV-associated disease. A more granular comparison (number and size of nodes, radiological extranodal extension) would require radiological review.

The magnitude of the prognostic effect of p16 (adjusted HR ≈ 3) is consistent with the survival gradient reported in RTOG 0129 and in recent meta-analyses [8,28]. Because age influences non-cancer mortality and differed between groups, we presented models with and without age; the p16 estimate was stable without age, but it was attenuated and imprecise in patients treated with curative intent (HR 2.30; 95% CI 0.99–5.33), a model with fewer than ten events per parameter. The association therefore cannot be regarded as independent of treatment allocation and baseline fitness. Nevertheless, OS captures deaths from second primaries and comorbidity, which are more frequent in heavy smokers and drinkers, and disease-specific survival would better isolate tumour biology. In addition, comorbidity and performance status—strong determinants of OS in an older cohort with substantial tobacco and alcohol exposure—were not available for adjustment. Because both are likely to be worse in p16-negative patients, the adjusted HR of 3.00 may be partly confounded by baseline health status and is best regarded as an upper estimate of the independent association of p16 status with OS. The attenuation observed in curatively treated M0 patients (HR 2.30), a group selected in part for fitness, is consistent with this; further adjustment for, or stratification by, treatment modality attenuated the estimate to 2.23 and 2.09, with confidence intervals including 1, suggesting that treatment allocation also explains part of the association. Cause of death was not recorded; linkage with the regional mortality registry to classify deaths as cancer-related or due to other causes would allow a competing-risks analysis in future work. Because follow-up was not updated to a common date while deaths were identified beyond the last clinical contact, censoring may have been informative; the association with p16 status was nevertheless present with administrative censoring at either an early common date (HR 4.45) or a late common date (HR 2.51).

Alcohol use differed markedly by p16 status but was not associated with OS after adjustment (HR 0.90), also when modelled in three categories. Two explanations are likely. First, alcohol use and p16 status were strongly correlated, so adjustment for p16 absorbs much of the prognostic information carried by drinking. Second, alcohol use was recorded only as current, former or never drinking, without quantity, so the binary ever/never variable in the model cannot distinguish light from heavy drinkers and may have diluted any dose-dependent effect. Because intake was not reliably quantified, a dose–response analysis was not possible, and this null result should not be interpreted as evidence that alcohol lacks prognostic relevance.

This study has several limitations. First, its retrospective single-centre design limits generalisability. Second, HPV-DNA testing was performed in only half of p16-positive tumours and was not applied to p16-negative tumours (except three), so discordance and the prevalence of p16-negative/HPV-positive tumours could not be fully estimated. In particular, without systematic testing of p16-negative tumours we could not identify p16-negative/HPV-positive cases, a recognised discordant group with distinct, intermediate prognosis [16], so our p16/HPV classification is incomplete and some p16-negative tumours may in fact be HPV-driven. Third, only DNA-based testing was used; E6/E7 mRNA detection is the reference standard for transcriptionally active HPV [17,29]. Fourth, follow-up is relatively short (median 28 months) and was not updated to a common cut-off date, and cause of death and recurrence dates were not available, precluding disease-specific and disease-free survival analyses. Fifth, tobacco exposure was not quantified in pack-years, and alcohol intake was recorded without quantitative data. Sixth, comorbidity and performance status were not available for adjustment, so residual confounding of the p16 association by baseline health status is possible. Seventh, p16 scoring was not centrally re-reviewed, inter-observer agreement was not assessed and three different HPV-DNA assays were used, so assay and interpretation variability may have contributed to the observed discordance. Eighth, the curative-intent models had fewer than ten events per parameter, second primary tumours were classified clinically without systematic molecular comparison with the index tumour, and case-level analytical data for the discordant tumours (tumour cellularity, assay and p16 staining pattern) could not be retrieved. Strengths include the consecutive, contemporary cohort staged uniformly with TNM-8, exclusion of p16-negative unknown primaries, explicit assessment of testing selection with a quantitative bias analysis, staging-independent nodal comparison, and survival analyses based on individual patient data with sensitivity analyses.

## 5. Conclusions

In northern Spain, approximately 37% of OPSCC are p16-positive, with no evidence of change in recent years; because p16 is only a surrogate, the HPV-attributable fraction is probably lower. p16-positive disease has a distinct clinical profile and better overall survival; the association persisted after adjustment for stage, alcohol, and age, but was attenuated and imprecise in curatively treated patients. Among tested p16-positive tumours, 21.4% lacked HPV-DNA; because testing was selective, sensitivity analyses gave a plausible range of 16–36%, and the direction of residual bias is uncertain. Systematic dual p16/HPV testing should be implemented in low-prevalence regions, ideally extending HPV testing to p16-negative tumours with a suggestive phenotype, before p16 status is used to guide prognosis, staging-driven treatment decisions, or de-escalation. This recommendation rests mainly on external evidence, including HNCIG-EPIC and the 2025 CAP guideline [16,19].

## Figures and Tables

**Figure 1:**
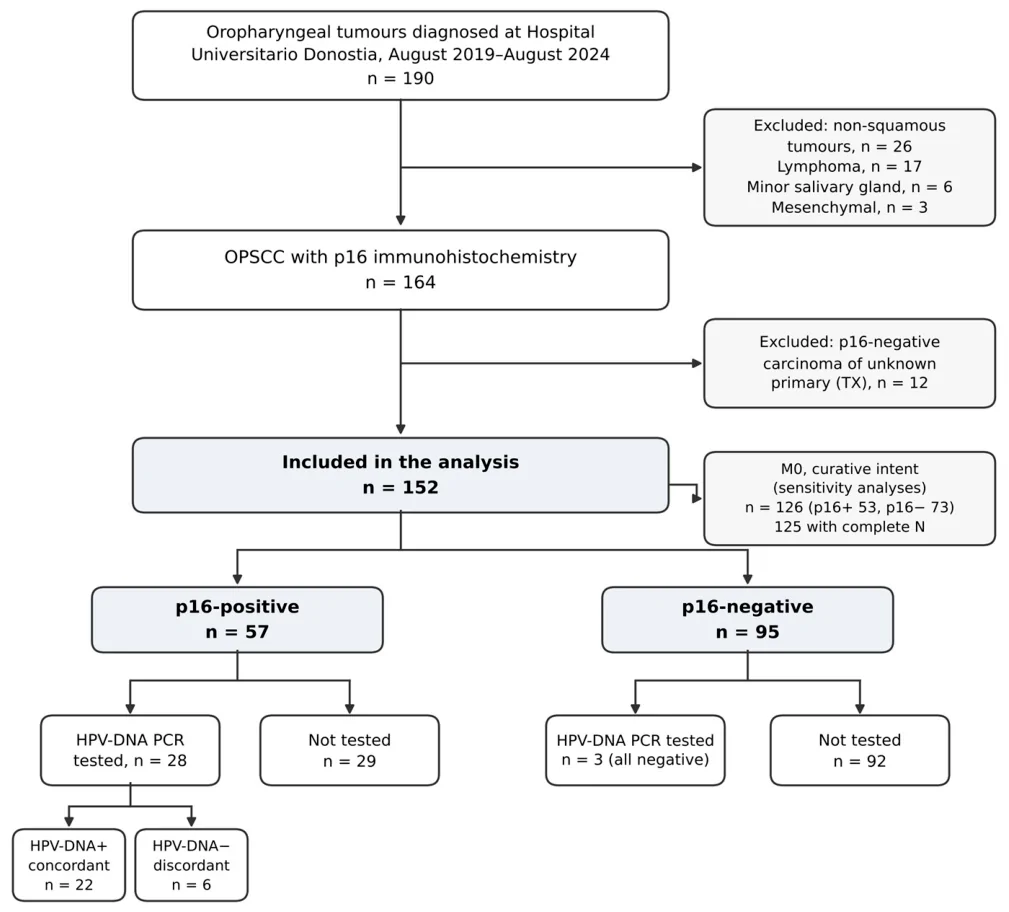
STROBE flow diagram of patient selection, HPV-DNA testing and the curative-intent cohort used in sensitivity analyses. OPSCC, oropharyngeal squamous-cell carcinoma.

**Figure 2:**
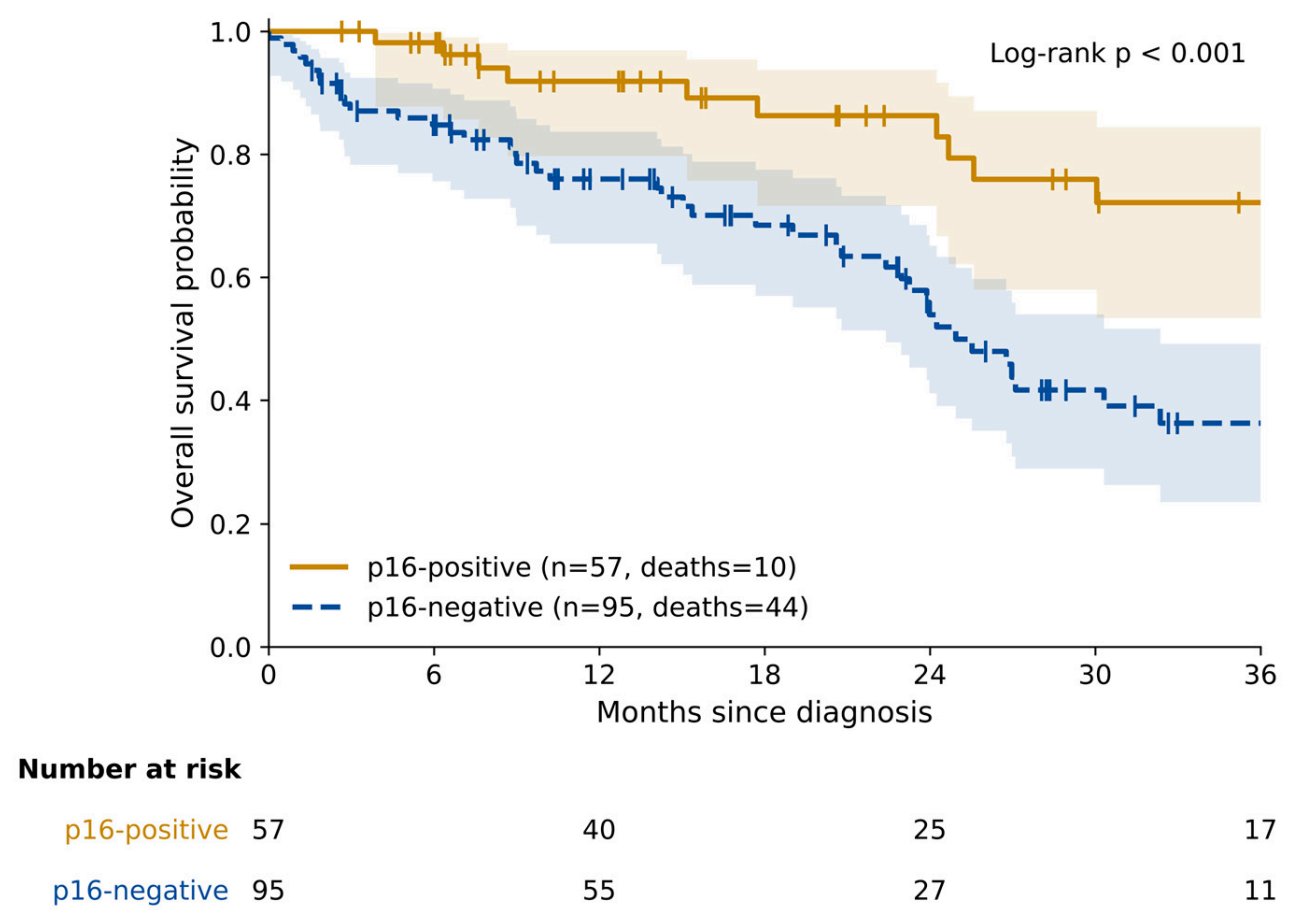
Kaplan–Meier overall survival according to p16 status. Curves are truncated at 36 months, when fewer than 20% of patients remained at risk. Shaded areas show 95% confidence intervals; vertical ticks indicate censored observations; numbers at risk are shown below the *x*-axis. Log-rank *p* < 0.001.

**Table 1:** Patient characteristics, treatment, and events according to p16 status (n = 152).

Variable	p16-Positive (n = 57)	p16-Negative (n = 95)	*p*
Age, years, mean ± SD (range)	63.6 ± 8.4 (42–84)	67.9 ± 8.4 (52–87)	0.003
Sex			
Male	39 (68.4)	60 (63.2)	0.60
Female	18 (31.6)	35 (36.8)	
Tobacco			
Current	24 (42.1)	58 (61.1)	0.060
Former	17 (29.8)	22 (23.2)	
Never	16 (28.1)	15 (15.8)	
Alcohol			
Current	19 (33.3)	49 (51.6)	0.001
Former	2 (3.5)	14 (14.7)	
Never	36 (63.2)	32 (33.7)	
Subsite			
Palatine tonsil	25 (43.9)	26 (27.4)	0.001
Base of tongue	25 (43.9)	32 (33.7)	
Soft palate	0 (0)	18 (18.9)	
Tonsillar pillar/pharyngeal wall	3 (5.3)	14 (14.7)	
Not specified	4 (7.0)	5 (5.3)	
Tonsil or base of tongue	50 (87.7)	58 (61.1)	<0.001
Primary treatment			
Chemoradiotherapy	29 (50.9)	42 (44.2)	0.003 *
Transoral surgery	23 (40.4)	20 (21.1)	
Radiotherapy alone	2 (3.5)	10 (10.5)	
Open surgery	0 (0)	1 (1.1)	
Systemic therapy alone	3 (5.3)	9 (9.5)	
Palliative/best supportive care	0 (0)	13 (13.7)	
Events during follow-up			
Recurrence	5 (8.8)	26 (27.4)	—
Distant metastasis	1 (1.8)	7 (7.4)	—
Second primary tumour	9 (15.8)	36 (37.9)	—
Death	10 (17.5)	44 (46.3)	<0.001

Values are n (%) unless stated. *p* values: *t* test for age; Fisher’s exact test for 2 × 2 comparisons; χ^2^ test otherwise; * Monte Carlo exact test. Event proportions are crude and were not compared statistically, because follow-up and competing mortality differed between groups and event dates were not available. Adjuvant treatment after transoral surgery: radiotherapy 5 and chemoradiotherapy 10 of 23 p16-positive patients; radiotherapy 4 and chemoradiotherapy 7 of 20 p16-negative patients; the patient treated with open surgery received adjuvant radiotherapy.

**Table 2:** Clinical staging (TNM-8) and staging-independent nodal descriptors.

Variable	p16-Positive (n = 57)	p16-Negative (n = 95)	*p*
cT (system-specific)			
T0	4 (7.0)	—	
T1	14 (24.6)	10 (10.5)	
T2	19 (33.3)	28 (29.5)	
T3	5 (8.8)	12 (12.6)	
T4 (p16+)/T4a (p16−)	15 (26.3)	31 (32.6)	
T4b	—	14 (14.7)	
T3–T4	20 (35.1)	57 (60.0)	0.004
cN (system-specific)			
N0	6 (10.5)	28 (29.5)	
N1	28 (49.1)	6 (6.3)	
N2 (p16+)/N2a–c (p16−)	19 (33.3)	35 (36.8) †	
N3 (p16+)/N3a–b (p16−)	4 (7.0)	25 (26.3)	
Staging-independent nodal descriptors			
Any nodal involvement (cN+)	51 (89.5)	66/94 (70.2)	0.008
Bilateral/contralateral nodes ‡	19 (33.3)	12 (12.6)	0.003
Any node > 6 cm §	4 (7.0)	1 (1.1)	0.066
Clinical extranodal extension (cN3b)	n.a.	24 (25.3)	—
cM1 at diagnosis	3 (5.3)	3 (3.2)	0.67

Values are n (%). N categories are not compared statistically because p16-positive and p16-negative OPSCC use different N definitions in TNM-8. † N2a 7, N2b 16, N2c 12; N stage missing in one patient. ‡ p16-positive cN2 or p16-negative cN2c. § p16-positive cN3 or p16-negative cN3a. n.a., not applicable.

**Table 3:** HPV-DNA testing in p16-positive tumours and comparison of tested versus untested patients.

Variable	Tested (n = 28)	Not Tested (n = 29)	*p*
HPV-DNA PCR result			
Positive (concordant)	22 (78.6)	—	
Negative (discordant)	6 (21.4; 95% CI 10.2–39.5)	—	
Genotype (available in 12 of 22 positive)			
HPV16 (one with HPV6 co-infection)	10	—	
HPV35	1	—	
HPV54	1	—	
Characteristics			
Age, mean	61.8	65.4	0.107
Female	11 (39.3)	7 (24.1)	0.263
Never-smoker	11 (39.3)	5 (17.2)	0.082
Never-drinker	23 (82.1)	13 (44.8)	0.006
Tonsil/base of tongue	24 (85.7)	26 (89.7)	0.706
T3–T4	7 (25.0)	13 (44.8)	0.167
N2–N3 (p16+ system)	10 (35.7)	13 (44.8)	0.592
M1	0 (0)	3 (10.3)	0.237

Values are n (%). Discordance = p16+/HPV-DNA− among tested. Three p16-negative tumours were also tested (all HPV-DNA-negative). Unspecified subsites are counted as other than tonsil/base of tongue.

**Table 4:** Case-level characteristics of the six discordant (p16+/HPV-DNA−) tumours and of the tumour-harbouring-only HPV54.

	D1	D2	D3	D4	D5	D6	HPV54-Only
Year of diagnosis	2020	2022	2022	2022	2024	2024	2023
Age (years)/sex	64/F	63/F	62/M	64/M	63/M	71/F	68/M
Subsite	Tonsil	BOT	Unspecified	Tonsil	Pharyngeal wall	Tonsil	Pharyngeal wall
Tobacco	Current	Current	Never	Never	Current	Current	Current
Alcohol	Never	Current	Never	Never	Former	Never	Current
cT/cN (TNM-8)	T1 N2a *	T4a N3b *	T4a N2c *	T4a N3b *	T1 N0 *	T1 N2b *	T1 N0
Specimen tested	US nodal biopsy	US nodal biopsy	US nodal biopsy	US nodal biopsy	Office biopsy	US nodal biopsy	Office biopsy
HPV-DNA result	Negative	Negative	Negative	Negative	Negative	Negative	HPV54 only
Primary treatment	TOS + adj CRT	TOS + adj CRT	CRT	CRT	TOS	CRT	TOS
Recurrence	No	Yes	No	Yes	No	No	No
Second primary	No	No	No	No	Yes	No	No
Vital status (FU, months)	Alive (41.3)	Died (30.1)	Died (17.7)	Alive (28.5)	Alive (7.6)	Alive (3.3)	Alive (12.7)

D1–D6, discordant tumours ordered by date of diagnosis. * Staged with the p16-negative TNM-8 classification in the clinical records. In node-positive cases, testing was performed on an ultrasound-guided biopsy of the cervical adenopathy; in the two cN0 cases, on a biopsy of the primary tumour obtained in the clinic under local anaesthesia. Tumour cellularity after macrodissection, assay, and p16 staining pattern could not be retrieved for individual cases because of the retrospective design. adj, adjuvant; BOT, base of tongue; CRT, chemoradiotherapy; F, female; FU, follow-up from diagnosis; M, male; TOS, transoral surgery; US, ultrasound-guided.

**Table 5:** Kaplan–Meier overall survival estimates (95% CI).

Group	1 Year	2 Years	3 Years	Deaths/n
p16-positive	91.9 (79.7–96.9)	86.3 (71.6–93.7)	72.2 (53.4–84.4)	10/57
p16-negative	76.0 (65.5–83.7)	53.9 (41.2–65.1)	36.3 (23.5–49.2)	44/95

Log-rank *p* < 0.001 (p16-positive vs. negative). Survival estimates for combined p16/HPV-DNA groups are not reported because of the very small numbers (see text).

**Table 6:** Cox-regression analysis for overall survival (152 patients, 54 deaths; multivariable models include the 151 patients with complete N data).

Variable	Univariable HR (95% CI)	*p*	Multivariable * HR (95% CI)	*p*
Age (per 10 years)	1.44 (1.03–2.03)	0.033	1.34 (0.97–1.87)	0.078
Female sex	0.64 (0.35–1.14)	0.13	—	
p16-negative vs. positive	3.56 (1.78–7.12)	<0.001	3.00 (1.44–6.26)	0.003
Current smoker vs. never	1.02 (0.53–1.95)	0.96	—	
Former smoker vs. never	1.12 (0.49–2.60)	0.79	—	
Alcohol ever vs. never	1.44 (0.82–2.54)	0.21	0.90 (0.50–1.62)	0.72
Subsite other than tonsil/BOT	1.74 (1.00–3.01)	0.050	—	
T3–T4 vs. T0–T2	2.32 (1.31–4.13)	0.004	1.96 (1.09–3.52)	0.025
N2–N3 vs. N0–N1	1.61 (0.92–2.80)	0.094	1.17 (0.66–2.09)	0.59
M1 at diagnosis	3.08 (1.11–8.55)	0.031	—	
Sensitivity analyses for p16-negative				
Model without age			3.20 (1.54–6.64)	0.002
M0, curative intent only (n = 125; 35 deaths)			2.30 (0.99–5.33)	0.052
Including p16− unknown primaries (n = 164)	3.78 (1.90–7.50)	<0.001		

* Pre-specified model: p16, T, N, alcohol, and age; likelihood-ratio χ^2^ = 25.0, *p* < 0.001; Harrell’s C = 0.70. The curative-intent model is adjusted for the same covariates. HR, hazard ratio; BOT, base of tongue. Unspecified subsites (n = 9) are grouped with other subsites; treating them as missing gave HR 1.66 (0.90–3.06). The curative-intent model had 35 deaths for five parameters (7.0 events per parameter) and is exploratory.

## Data Availability

The data presented in this study are available on reasonable request from the corresponding author because of patient privacy restrictions. Analysis scripts are available from the corresponding author on request.
